# Advance Care Planning in Patients with Primary Malignant Brain Tumors: A Systematic Review

**DOI:** 10.3389/fonc.2016.00223

**Published:** 2016-10-24

**Authors:** Krystal Song, Bhasker Amatya, Catherine Voutier, Fary Khan

**Affiliations:** ^1^Department of Rehabilitation Medicine, Royal Melbourne Hospital (RMH), Melbourne, VIC, Australia; ^2^Department of Medicine (RMH), University of Melbourne, Melbourne, VIC, Australia; ^3^School of Public Health and Preventive Medicine, Monash University, Melbourne, VIC, Australia

**Keywords:** advance care planning, end-of-life care, communication, glioma, brain tumor

## Abstract

Advance care planning (ACP) is a process of reflection and communication of a person’s future health care preferences, and has been shown to improve end-of-life (EOL) care for patients. The aim of this systematic review is to present an evidence-based overview of ACP in patients with primary malignant brain tumors (pmBT). A comprehensive literature search was conducted using medical and health science electronic databases (PubMed, Cochrane, Embase, MEDLINE, ProQuest, Social Care Online, Scopus, and Web of Science) up to July 2016. Manual search of bibliographies of articles and gray literature search were also conducted. Two independent reviewers selected studies, extracted data, and assessed the methodologic quality of the studies using the Critical Appraisal Skills Program’s appraisal tools. All studies were included irrespective of the study design. A meta-analysis was not possible due to heterogeneity amongst included studies; therefore, a narrative analysis was performed for best evidence synthesis. Overall, 19 studies were included [1 randomized controlled trial (RCT), 17 cohort studies, 1 qualitative study] with 4686 participants. All studies scored “low to moderate” on the methodological quality assessment, implying high risk of bias. A single RCT evaluating a video decision support tool in facilitating ACP in pmBT patients showed a beneficial effect in promoting comfort care and gaining confidence in decision-making. However, the effect of the intervention on quality of life and care at the EOL were unclear. There was a low rate of use of ACP discussions at the EOL. Advance directive completion rates and place of death varied between different studies. Positive effects of ACP included lower hospital readmission rates, and intensive care unit utilization. None of the studies assessed mortality outcomes associated with ACP. In conclusion, this review found some beneficial effects of ACP in pmBT. The literature still remains limited in this area, with lack of intervention studies, making it difficult to identify superiority of ACP interventions in pmBT. More robust studies, with appropriate study design, outcome measures, and defined interventions are required to inform policy and practice.

## Introduction

Primary brain tumors (BT) are a diverse group of neoplasms, affecting approximately 7 persons per 100,000 population annually worldwide (1). Primary BT predominantly are malignant gliomas, and half of these are glioblastoma multiforme (GBM) (1). The diagnosis of primary malignant brain tumor (pmBT) heralds a dramatic change in life expectancy for patients, with limited effective treatment options, progressive neurological deterioration, and high mortality rates. Despite advances in available therapies, GBM patients have a median survival of approximately 14 months only (2). Many pmBT patients experience ongoing difficulties related to the disease itself with high symptom burden (3, 4), and treatments used. They also experience other disabilities, such as fatigue, difficulties with mobility and self-care, cognitive and intellectual decline, behavioral dysfunction, personality changes, and psychological problems, such as depression and anxiety. There are significant implications of these disabilities on caregivers with role reversal within families, vocational issues, financial strain, and reduced quality of life (QoL) (5, 6).

The neuropalliative supportive care needs of pmBT patients had previously been recognized and national guidelines acknowledged ongoing considerable gaps in provision of end-of-life (EOL) care, including earlier Advance Care Planning (ACP) discussions for those with terminal conditions (7–9). ACP is the ongoing process that involves decisions made by patients, in consultation with surrogate decision-makers, family and health care providers regarding their values, beliefs, life-sustaining treatment preferences, goals of care (GOC), and palliative care options, should they later become incapable of expressing such wishes. ACP may further include the patient completing an advance directive (AD) which documents his or her wishes and/or appointment of a substitute decision-maker.

Advance care planning is recognized as an important element in improving EOL care, and one that is increasingly used in an international context because it allows patients to direct their own ongoing care. This contributes to shared decision-making, resulting in enhanced QoL for the patient. ACP has also been shown to increase patient and family satisfaction with EOL care (10, 11) and improve understanding and compliance between physicians and family members with patients’ wishes for EOL care (10, 12). In addition, ACP increases the likelihood of a person dying in his or her preferred place (13), increases hospice use, and reduces hospitalization, with lower stress levels, anxiety, and depression in surviving relatives (10, 11).

Discussing EOL issues with pmBT patients is especially challenging. The rapidity of cognitive decline due to tumor growth, tumor-related seizures or treatment effects (14) often precludes the expression of future wishes and treatment preferences, leaving families and clinicians to decide whether and when to withdraw interventions after patients have lost capacity to decide for themselves. Furthermore, delirium, dysarthria, dysphasia, and personality changes are also common (3, 8), which may lead to impaired communication about complex topics, such as hydration, nutrition, steroid interruption, and palliative sedation (3). Many often have limited understanding of their treatment options, and prognostic uncertainty impacting on this process, resulting in difficulty achieving realistic expectations and determining appropriate treatment decisions (15). Cultural, religious and spiritual factors also tend to play a role in EOL decision-making. Many clinicians often avoid the topic in practice due to time pressures, lack sufficient communication skills training, and are uncertain about timing and content of ACP with fear of upsetting patients (16).

Advance care planning has been widely explored in the literature in different patient cohorts, including cancer, chronic renal failure, heart failure, chronic obstructive pulmonary disease, and other life-limiting conditions. A number of studies had previously investigated ACP in the BT cohort (17, 18). The body of research in this area is growing; however, published studies vary in methodology, scope, and interventions with different findings and conclusions. The outcomes following ACP interventions need to be established. However, to date, there is no systematic review evaluating ACP in pmBT patients to guide clinicians in ACP implementation. Therefore, the aim of this study is to systematically evaluate the current evidence and efficacy of ACP in pmBT patients to assist treating clinicians, and to ensure that services and supports are directed appropriately in this population.

## Methods

An integrated approach was employed, which included a comprehensive review of literature (peer review and gray literature), using medical and health science electronic databases (PubMed, Cochrane, Embase, MEDLINE, ProQuest, Social Care Online, Scopus, and Web of Science) up to July 2016. Bibliography search of identified articles and manual search of relevant journals for additional references were conducted. A further gray literature search was conducted using different internet search engines and websites, such as the PQDT Open, OATD, and Google Scholar. In addition, various healthcare institutions; and governmental and non-governmental organizations associated with management of individuals with pmBT were also explored for relevant studies. Authors and known experts in the field were contacted.

The search strategy used Medical Subject Headings (MeSH) or equivalent key words relating to pmBT and ACP. Premutations of the following search terms were truncated and exploded: brain cancer, neoplasm or malignancy, ACP, ADs, EOL communication, life-sustaining treatment preferences, EOL decision-making, EOL care, and living will. All unpublished studies and all trials registered in relevant registries were scrutinized to reduce publication bias.

### Inclusion and Exclusion Criteria

A protocol for searching was established prior to commencement of the search. ACP was defined as *“any advance discussions or directives pertaining to patients’ life-sustaining treatment preferences, goals of care, palliative care options, appointment of a health care proxy and may further include the completion of an AD*” (19). Inclusion criteria were determined as follows: (1) articles published in peer-reviewed journals, irrespective of study design; (2) studies with participants with confirmed diagnosis of pmBT [also specified as malignant glioma, high-grade glioma (HGG), anaplastic astrocytoma, GBM, anaplastic oligodendroglioma, oligoastrocytoma or World Health Organization (WHO) tumor grades III and IV]; and (3) 18 years and above. Other studies conducted in different patient cohorts were also included if they provided data for >50% of pmBT patients.

Articles were excluded if the participant subgroups with pmBT were not clearly defined. Theses, narrative reviews, editorials, case reports/series, and published conference abstracts were also excluded.

### Study Selection and Data Extraction

Two authors (Krystal Song and Fary Khan) independently screened all identified study titles and abstracts for inclusion based on the selection criteria. Any disagreements were resolved by consensus discussion with the third author (Bhasker Amatya). Preliminary synthesis was conducted by using a standard *pro forma* created *a priori* with data extraction from studies, which included study characteristics (publication year and country, study type, sample characteristics, methodology, outcome measures, key findings) and intervention characteristics where feasible [type, intensity, domain, setting, delivery mode, duration and any information available about training and feasibility (e.g., adherence)]. Additional description of interventions was obtained from the study corresponding author where necessary.

Thematic analysis of the primary findings presented by each study was undertaken. Findings were individually scrutinized by the two authors (Krystal Song and Fary Khan), who discussed, extracted, and refined key emergent themes. Final themes were then developed and reviewed with feedback sought from the third author (Bhasker Amatya). Any disagreements were resolved by group discussions.

Two authors (Krystal Song and Bhasker Amatya) independently assessed the methodologic quality and grade of evidence of included studies with the CASP tool (20). The CASP tool uses a systematic approach to appraise different study designs from the following domains: study validity, methodologic quality, presentation of results, and external validity (20). The articles were graded independently, and any disagreements were resolved through consensus. Each of the items from the checklists were judged with “yes” (low risk of bias, score 1), “no” (high risk of bias), or “cannot tell” (unclear or unknown risk of bias, score 0). Total scores were used to grade the methodologic quality of each study assessed [maximum score 11 for randomized controlled trial (RCTs); 12 for cohort studies; 10 for qualitative studies] (20).

## Results

### Results of the Search

**Figure 1:**
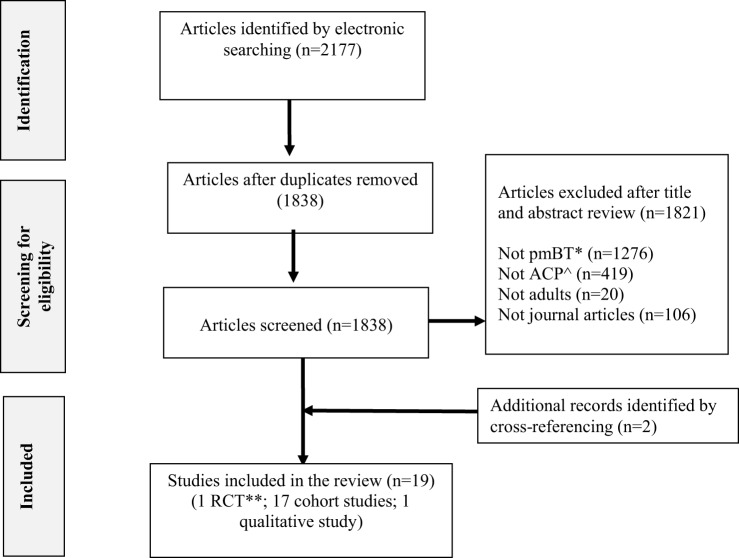
**Flow chart of review selection process**. ^ACP, advance care planning; *pmBT, primary malignant brain tumor; **RCT, randomized controlled trial.

The combined searches retrieved a total of 2177 published titles and abstracts, of which 1838 were screened after removal of duplicates. Seventeen articles met inclusion criteria for analysis. Further citation and journal hand searches identified two articles that were included for final analysis. In total, 19 articles (1 RCT, 17 cohort studies, 1 qualitative study) were determined as eligible for this systematic review. See Figure 1 for a PRISMA flow diagram of the study selection process.

### Study Characteristics

The included studies were conducted in different countries: three each were conducted in the USA, Italy, and Australia; two each in Germany, Austria, Netherlands, and UK, while two studies were multicentered and conducted in three different countries: The Netherlands, Austria, and UK. The included studies evaluated a total of with 4686 participants. Detailed information regarding the included studies is presented in Table 1. Only one randomized controlled trial (RCT) was identified which evaluated a video decision support tool on ACP in pmBT patients (17). Other were observational studies, including 14 retrospective and 3 prospective cohort studies and 1 was a qualitative study.

**Table 1:** **Summary of included studies**.

	Reference, country	Aim of study	Study type	Methodology	Participants and demographic characteristics	Key findings
1.	Arber et al. (6), UK	To investigate symptom profile and access to rehabilitation and supportive care services of primary malignant brain tumor (pmBT) patients and carers	Retrospective cohort study	Data collected included demographic information, symptom profile, care issues, services used, and place of death (POD)	*N* = 70 Mean age 64 years (range 21–94); women 44%; glioblastoma multiforme (GBM) 59%	79% (*n* = 55) had details of POD including 30% who died at hospice, 16% at home, 20% in another hospital, 13% nursing home
2.	Collins et al. (21), Australia	To investigate the clinical presentation, patterns of supportive and palliative care (PC) utilization for short-term malignant glioma patients (survival time <120 days)	Retrospective cohort study	Retrospective cohort study of incident malignant glioma cases between 2003 and 2009 surviving <120 days in Victoria, Australia. Data collected included clinical symptoms, hospital utilization, supportive care utilization, PC involvement, and POD	*N* = 482 (no. of shorter term survivors of total 1,160 patients with incident PMG diagnosis) Group 1 (*n* = 218, 19%) died during diagnosis admission; 89% GBM; mean number of hospital bed days = 40 (median 33) Group 2 (*n* = 264, 23%) survived the diagnosis admission but died within 120 days from diagnosis; 86% GBM; mean number of hospital bed days = 17 (median 11); POD: home 32%, PC bed/hospice 38%, and acute hospital 30%	62% of Group 1 was admitted to a PC bed during the diagnosis admission. 22% of the cohort died without any PC contact Only 12% of Group 2 patients had a PC consultation for appropriate planning and support during their diagnosis admission as compared to 78% of group 1 patients
3.	Diamond et al. (22), USA	To evaluate the frequency of and characteristics associated with early vs. late hospice referral in pmBT patients	Retrospective cohort study	Data collected included demographic data, presence of health care proxy (HCP), clinical characteristics and utilization of homecare services, and characteristics associated with late vs. earlier referral to home hospice	*N* = 160 Mean age 63.4 years (SD, 15.6); women, 42%; 65% GBM; POD: home 82%, affiliated hospice facility 18%	Of 160 patients with pmBT followed to death in hospice care, 32 (22.5%) were enrolled within 7 days of death. In comparison to early hospice referral, a greater proportion was bedbound at admission (97.2 vs. 61.3%), aphasic (61.1 vs. 20.2%), unresponsive (38.9% vs. 4%) or dyspneic (27.8% vs. 9.7%). Male patients, Medicaid insurance coverage and those without a HCP were significantly associated with late referral. 36% (*n* = 58) of all patients had no appointed HCP at time of home hospice admission. 55.6% (*n* = 20) of those referred late to hospice did not have a HCP
4.	El-Jawahri et al. (17), USA	To evaluate the effectiveness of the use of goals-of-care (GOC) video to supplement a verbal description in improving end-of-life (EOL) decision-making for malignant glioma patients	Randomized controlled trial	50 participants with malignant glioma were randomly assigned to either a verbal narrative of GOC options at the EOL (control) (*n* = 27), or a 6-min video after the same verbal narrative (intervention) (*n* = 23) *Primary outcome measure*: post-intervention preference for EOL care *Secondary outcome measures*: change in knowledge scores after the narrative or video; decisional conflicts scores between both interventions	*N* = 50 Mean age, 54 years; range, 32–77 years; 44% women; 82% married; 76% had an advance directive (AD); 72% GBM	After verbal description, 25.9% of participants’ preferred life-prolonging care, 51.9% basic care and 22.2% comfort care. In the video arm, no participants preferred life-prolonging care, 4.4% preferred basic care, 91.3% preferred comfort care, and 4.4% were uncertain (*p* < 0.0001). The mean increase in knowledge score was higher in the video group compared to the verbal control group (1.9 vs. 0.9; *p* = 0.004). The mean uncertainty score regarding decision-making (range, 3–15; higher score indicating less uncertainty) was higher in the video group than in the verbal group (13.7 vs. 11.5, respectively; *p* < 0.002). 82.6% in the intervention arm reported being very comfortable watching the video
5.	Faithfull et al. (23), UK	To explore the referral and carer characteristics, illness trajectory, symptoms and services provided to carers and patients with pmBT referred to PC	Retrospective cohort study	Data collected included demographics, PC, service utilization, symptom profile and time from diagnosis of brain tumor to referral and death	*N* = 39	Out of 37 who died, POD: 33% (*n* = 13) home, 33% (*n* = 13) hospice, 13% (*n* = 5) nursing home
6.	Flechl et al. (24), Netherlands	To evaluate the caregivers’ experiences on the EOL phase of deceased GBM patients	Retrospective questionnaire completion by caregivers	Data collected included the caregiver’s view of the patients’ terminal phase, experiences and emotions of caregiver during the last three months of patients’ life, patients’ quality of life (QoL), quality of care, EOL preferences and POD information	*N* = 52. Mean age, 63 years; range, 35–83 years; women, 37%; POD: home 40%, hospice 12%, hospital 46%, and nursing home 2%	30/52 patients had expressed preferences for their place of death; 79% wished to die at home and 68% of them fulfilled this. No patient had expressed a formal AD
7.	Gofton et al. (25), USA	To evaluate the PC needs and EOL decisions of patients with primary and metastatic brain tumors	Retrospective cohort study	Data collected included evidence of resuscitation at the EOL, evidence of cancer directed therapy in the last month of life, HCP, PC consultation, hospice discussion, and evidence of discussion regarding resuscitation wishes	*N* = 168 (total sample) including primary BT (*n* = 144) or metastatic brain tumor (*n* = 24) *Grade III/IV glial tumors: N* = 101 (70.1% of primary brain tumor sample); Mean age, 58.2 ± 15, 42 (41.6%) women; 43 (42.6%) deceased; POD (*n* = 43): home 20.9%, outpatient hospice support 34.9%, hospice 32.6%, and nursing home 2.3%	Of the WHO grade III/IV brain tumor patients who died during the course of the study (*n* = 43), approximately 12% had no documented EOL discussions, 76.7% had an appointed HCP, and 65.1% had a do-not-resuscitate order. The timing of EOL discussions to death such as hospice planning (1–140 days) (median = 28 days) and resuscitation wishes (1–140 days) (median = 31.5 days) varied widely
8.	Golla et al. (26), Germany	To evaluate whether GBM patients are capable of regular self-assessment of their symptoms and needs during disease progression	Prospective cohort study	GBM patients’ PC issues were assessed from diagnosis to death or for at least 12 months every 7 weeks (±8 days). Each assessment consisted of two parts: (1) semi-structured interview regarding current disease status, treatment, burdensome symptoms, EOL care wishes; (2) PC assessments using the Hospice and Palliative Care Evaluation (HOPE, 27 items) and the Palliative Outcome Scale (POS, 11 items)	*N* = 13 Age range, 45–71 years; women, 31%; 15% (*n* = 2) AD, 8% (*n* = 1) HCP; POD: home 23%, hospice 31%, nursing home 8%, and hospital 38%	31% of patients in this study obtained AD during the course of GBM disease, and 62% obtained HCPs. Repeated interviews in the current study may have possibly influenced GBM patients’ ACP
9.	Heese et al. (27), Germany	To evaluate caregivers’ perception of medical and psychological support received during the final disease phase of glioma patients	Retrospective cohort study	Data collected from caregivers through questionnaires included patients’ place of PC during final 4 weeks and POD, supporting physician, medical support for EOL problems, receipt of counseling by physician with regards to PC	*N* = 605 WHO Grade IV tumor 71.3% (*n* = 398); mean age 58.8 years (range 17.6–86.7 years) (total sample); POD: home 47.7%, hospice 19.3%, nursing home 7.3%, and hospital 22.6%	Medical support was provided by GPs in 72.3% of cases, 29.9% by physicians affiliated with a nursing home or hospice, 17% by general oncologists and 6% by specialized neurooncologists. 21.3% of patients received care from physicians who specialized in PC medicine
10.	Koekkoek et al. (28), Netherlands, Austria, UK	To evaluate cross-national differences whether different patterns of EOL care meet patient’s specific needs	Retrospective cohort study	Data collected from relatives of deceased pmBT patients examined: (1) EOL care organization such as place of care, transitions to another health care setting, and POD; (2) treatment preferences, including presence of AD and preferred POD; (3) experiences with EOL care: actual POD, quality of information provided by treating physician, satisfaction with explanation of treatment decisions and symptoms treatment; and (4) perceived quality of care (QOC) during last 3 months before death	*N* = 207 Total number of patient’s relatives who could be traced: Dutch (*n* = 131), Austrian (*n* = 119) and British (*n* = 89). Returned questionnaires included 83 (63%) Dutch, 72 (61%) Austrian, and 52 (58%) British relatives	Three months before death, 75% of patients were at home. POD differed significantly (*p* < 0.001). In the Netherlands, Austria and UK, respectively, patients most often died at home (60%), in a hospital (41%) or hospice (41%) (*p* < 0.001). Three months before death, patients expressed specific treatment wishes to their relatives in almost 80% in Dutch population, in contrast to 58 and 48% of Austrian and British patients (*p* < 0.001) Written AD was used in 46% of Dutch, 37% of British, but only 6% of Austrian patients (*p* < 0.001). In 10% of patients, physicians made decisions that were incongruent with patient’s earlier expressed preferences, irrespective of country. Dying at the preferred place, satisfaction with information provided and effective symptom treatment were independently associated with good QOC
11.	Koekkoek et al. (29), Netherlands, Belgium, Austria	To evaluate prevalence of symptoms and medication management in pmBT patients during EOL phase	Retrospective cohort study	Data collected included symptoms, general EOL symptoms, perceived QOC	*N* = 178 Mean age 59.7 years; women 29.8%; Grade IV brain tumor 89.3%	POD included home 54.7%, hospice 18.2%, hospital 12.4%, nursing home 12.4%
12.	Pace et al. (3), Italy	To explore EOL issues and treatment decisions in a population of brain tumor patients followed at home until death by a neuro-oncologic home care palliative unit	Retrospective cohort study	Patients were enrolled in a comprehensive program of neuro-oncological home care, receiving neurological assistance, nursing, psychosocial support, and rehabilitation at home. Data collected included clinical symptoms, PC treatments, and EOL treatment decisions	*N* = 169 Mean age, 56 years; range, 15–93 years; women, *n* = 87; 79.9% GBM	Only 6% had early AD about EOL treatment. Majority were not competent to make treatment decisions in the last month of life. Tube feeding was installed in 13% of patients, steroids tapered in 45% of patients and palliative sedation in 13% of patients. Only 52.9% of patients were fully aware about prognosis, 27% partially aware, and 20% unaware
13.	Pace et al. (30), Italy	To evaluate a pilot neuro-oncological home care program of comprehensive palliative care for brain tumor patients	Retrospective cohort study	This model aimed to meet patients’ needs for care in all stages of disease, support the families and reduce the rehospitalization rate. The intensity of care changes in different stages of disease from low intensity (e.g., home visit, phone contact) to medium level intensity in the disease progression stage [more than one weekly visit, nursing assistance, psychological assistance, palliative advance care planning (ACP), and high intensity in the EOL stage (e.g., 3 weekly home visits)]. Data analyzed included POD, caregiver satisfaction, rehospitalization rate and the impact on costs to the health system	*N* = 848 Male/female ratio 439:409; mean age 57 years (range: 17–89 years); 50.1% GBM Of the 529 patients who died, 323 (61%) were assisted at home until the EOL, 117 (22.2%) died in hospital, and 89 (16.8%) died in hospice	Common EOL treatment decisions in 323 BT patients who were assisted at home until death included steroid withdrawal (45%), mild hydration (87%), tube feeding (13%) and palliative sedation (11%). Only 6% of these patients had an AD Cost-effective analyses performed in a subgroup of GBM patients showed that a group of patients assisted by the home care program (*n* = 72) had significantly lower hospital readmission rates and ICU utilization in the last 2 months of life than the control group (*n* = 72), who did not receive home assistance (16.7 vs. 38%, respectively; *P* < 0.001). There was also a statistically significant difference in economic cost for care delivered during hospital stays in the same period ($9990 vs. $76,000; *p* < 0.001)
14.	Pompili et al. (31), Italy	To evaluate the results of home PC and EOL issues in GBM patients	Retrospective cohort study	The intensity of the home care program ranged from low intensity (weekly home access or contact by phone standard ambulatory consultation) to a medium level of intensity in patients with more progressive stage (more than one weekly access, nursing assistance, psychological assistance, palliative ACP) and a high level of intensity of care in the EOL stage (at least 3 weekly accesses)	*N* = 122 100% GBM patients; 52.4% died (*n* = 64), median survival 13.34 months; POD: home 53.1%, hospice 34.4%, and 12.5% hospital	Only 6% of patients in this study had an AD
15.	Sizoo et al. (32), Netherlands	To evaluate the proportion of pmBT patients dying with dignity as perceived by their relatives; to identify disease and care factors correlated with dying with dignity	Retrospective survey of carers’ perspectives	Data collected included patients’ symptom profile, health-related QoL, decision-making, place and quality of EOL care, and dying with dignity	*N* = 81 Mean age at death, 61 years; range, 20–86 years; women, 36%; POD: home 57%, hospice 17%, nursing home 12%, and hospital 10%	75% of relatives of patient cohort indicated that the patient died with dignity. These patients had fewer communication deficits, fewer transitions between health care settings in EOL phase and more frequently died at preferred POD. Higher satisfaction rates with the physician, the ability to communicate EOL decisions and absence of transitions between settings were most predictive of a dignified death. Patients who died at home died most often with dignity (83%), followed by hospice (71%), hospital (63%), and nursing home (50%) patients (*p* = 0.255)
16.	Sizoo et al. (18), Netherlands	To evaluate the EOL decision (ELD) making process in high-grade glioma (HGG) patients	Retrospective cohort study	Questionnaire data collection from physicians and relatives regarding EOL conditions (patients’ ELD preferences, patients’ competence) and ELD-making (forgoing treatment and the administration of drugs with a potential life-shortening effect)	*N* = 101 Mean age at death, 60 years; range, N/A; women, 28%; Grade 4 HGG 88%, Grade 3 HGG 12%; POD: home 66%, hospice 10%, hospital 7%, and nursing home 16%	Of 101 patients, there was a 62% (*n* = 51) physician response rate, with relatives’ data for an additional 50 patients. 52% of patients were relatively incompetent to make decisions due to EOL cognitive deficits. 40% of patients did not have EOL preference discussions with their physician. Only 3% of patients declined this discussion. At least one EOL decision was made in 72% of patients, most often steroid withdrawal. In a subset of deceased patients (*n* = 50), 42% of patients had an AD, with 4% of physicians not being aware of this. Incongruence existed between patients’ EOL wishes with physician decisions in EOL care
17.	Sundararajan et al. (33), Australia	To evaluate the association between symptoms, receipt of supportive and PC, and POD of pmBT cases who survived for at least 120 days between their first hospitalization and death	Retrospective cohort study	Data collected included patient characteristics, receipt of supportive and PC, and POD. PC would include tasks of care education, clear information, emotional support and equipment access, carer support, exploration of preferences and informed decision-making	*N* = 678 Mean age 62y; women, 40%; median survival 11 months; GBM, 84%; POD: home 26%, hospice 49%, and acute hospital 25%	Increased receipt of PC consultation from 5 to 63% and the use of a hospice bed from 2 to 49% from diagnosis to hospitalization during which they died was shown. Patients having one or more symptoms were more than five times as likely to receive PC. Factors associated with POD were patients who received any PC in the last 120 days before death
18.	Song et al. (23), Australia	To evaluate the awareness and experience of brain tumor patients in discussing ACP	Qualitative prospective cohort study	Initial open-ended questionnaire followed by semi-structured interview questions explored ACP with primary and metastatic BT patients in a hospital and community setting. ACP discussions were analyzed using thematic analyses and grounded theory	*N* = 18 Mean age 51 years (range 22–65 years); women 61%; 56% (*n* = 10) GBM patients; 0% AD	Study findings showed that participants reported overall good QoL. Thematic analyses indicated that participants had limited awareness and understanding of ACP. There were variable views on appropriate timing of ACP discussions amongst participants and most felt that a medical facilitator of the decision-making process would be preferred
19.	Thier et al. (34), Austria	To investigate the signs, symptoms, and treatment strategies in GBM patients in the EOL phase	Retrospective cohort study	Data collected included signs, symptoms, and therapeutic strategies and was descriptively analyzed. Treatment decisions were discussed whenever possible with the patient and/or with proxies if available	*N* = 57 Women 32%; mean age at death, 50 years; mean survival, 48 weeks	With treatment strategies, 95% received opioids, 77% NSAIDs, 75% anticonvulsants, and 56% steroids. Only 2 (4%) patients had an AD

*pmBT, primary malignant brain tumor; POD, place of death; GBM, glioblastoma multiforme; PC, palliative care; HCP, health care proxy; GOC, goals-of-care; EOL, end-of-life; AD, advance directive; QoL, quality of life; QOC, quality of care; ELD, EOL decision; HGG, high-grade glioma*.

### Study Quality Assessment

There was consensus agreement amongst reviewers regarding methodology used in the studies. Table 2 provides a quality assessment of included studies using the CASP tool (20).The methodologic quality of included studies varied. The only included RCT was of moderate quality (CASP = 8/11). This study was, however, underpowered and had insufficient blinding procedures (17). The CASP scores for the 17 cohort studies ranged from 2 to 7 out of 12. The overall quality of most included cohort studies was assessed as low to moderate, of which eight studies were of moderate quality (3, 21, 22, 25, 28, 30, 32, 33). All cohort studies had substantial flaws in methodologic design with a high risk of bias related to heterogeneous patient characteristics, study design, and outcome analysis. Outcome measurement tools varied among studies, and some tools used were not validated.

**Table 2:** **Levels of quality of individual studies (CASP approach^a^)**.

Randomized controlled trials													

Study	Clear focused issue	Adequate randomization procedure	Participants properly accounted	Blinding of participants/assessors	Groups similar at start	Groups treated equally	Large treatment effect	Precise treatment effect	Applicability of results to local population	Clinically important outcomes considered	Benefits worth harm and costs		CASP Grade
El-Jawahri et al. (17)	+	+	+	−	−	+	+	+	+	+	?		8/11

**Cohort studies**													

**Study**	**Clear focused issue**	**Appropriate method**	**Appropriate cohort recruitment**	**Exposure accurately measured**	**Outcome accurately measured**	**Important confounding factors accounted**	**Adequate follow-up**	**Strong exposure and outcome relation**	**Precise results**	**Believe the results**	**Applicability of results of local population**	**Comparability of results with other available evidence**	**CASP Grade**

Arber et al. (6)	+	+	+	−	−	−	−	−	−	−	−	−	3/12
Collins et al. (21)	+	+	+	+	+	−	+	−	?	?	+	?	7/12
Diamond (22)	+	+	+	+	+	−	?	+	+	?	−	−	7/12
Faithfull et al. (24)	+	+	+	−	−	−	−	−	−	−	−	−	3/12
Flechl et al. (5)	+	+	−	−	−	−	−	−	−	−	−	−	2/12
Gofton et al. (25)	+	+	+	−	−	−	+	−	+	+	−	?	6/12
Golla et al. (26)	+	+	−	−	+	+	−	?	?	?	−	−	4/12
Heese et al. (27)	+	+	+	−	−	−	?	−	−	−	−	−	3/12
Koekkoek et al. (28)	+	+	+	+	−	+	?	+	+	−	−	−	7/12
Koekkoek et al. (29)	+	+	+	−	−	−	−	−	−	−	−	−	3/12
Pace et al. (30)	+	+	+	−	−	−	+	−	−	+	−	−	5/12
Pace et al. (30)	+	+	+	+	−	−	+	?	−	−	−	−	5/12
Pompili et al. (31)	+	+	+	−	−	−	−	−	?	−	−	−	3/12
Sizoo et al. (32)	+	+	+	−	−	+	?	+	+	+	−	?	7/12
Sizoo et al. (18)	+	+	+	−	−	−	?	−	−	−	−	−	3/12
Sundararajan et al. (33)	+	+	+	+	+	−	+	−	?	?	+	?	7/12
Thier et al. (34)	+	+	+	−	−	−	−	−	−	−	−	−	3/12

**Qualitative studies**													

**Study**	**Clear statement of aims**	**Appropriate qualitative methodology**	**Appropriate research design**	**Appropriate recruitment strategy**	**Appropriate data collection**	**Relationship between researcher and participants**	**Ethical issues taken into consideration**	**Sufficient data analysis rigor**	**Clear statement of findings**	**Research value**			

Song (23)^b^	+	+	−	−	+	−	+	−	?	+			5/10

*+, yes; −, no; ?, cannot tell*.

*Ratings: 8 or > out of 10: good quality; 5–7: moderate quality; <5: poor quality*.

*^a^CASP critical appraisal tool for qualitative research (20)*.

*^b^As this study was conducted by the author, critical appraisal of this study was performed by an independent reviewer (Professor Mary Galea)*.

### Synthesis of Results

Pooling of data was not possible due to heterogeneity among the included studies in terms of study design, interventions used and presentation of findings. Therefore, study findings are described narratively in most relevant subheadings.

#### Description of ACP Interventions

In a RCT, El-Jawahri et al. evaluated the feasibility and effectiveness of a video GOC decision support tool in facilitating ACP in pmBT patients (17). This video tool was used to supplement a verbal description in aiming to improve EOL decision-making in pmBT patients. Fifty participants were randomly assigned to either the verbal narrative (*n* = 27) or additional video tool after the verbal narrative (*n* = 23). The findings suggested that the video tool was effective in promoting comfort care and gaining confidence in decision-making compared to verbal description only. No participants in the video tool group preferred life-prolonging care, 4.4% preferred basic care, 91.3% preferred comfort care, and only 4.4% were uncertain (*p* < 0.0001), while in participants in the verbal narrative group, 25.9% of participants preferred life-prolonging care, 51.9% basic care, and 22.2% comfort care (17). However, the effect of the video intervention on QoL and care at the EOL were unclear. This study was underpowered with small sample size and has methodological flaws such as lack of blinding of assessors (17).

Another intervention study assessed the impact of a pilot program of comprehensive palliative care (including the provision of palliative ACP) for pmBT patients on place of death (POD), rehospitalization rate in the last months of life, and the cost-effectiveness of the care model (30). Of the 529 patients who died, 61% were assisted at home until the EOL, 22.2% died in hospital, and 16.8% died in inpatient hospice. Hospitalization readmission rates and intensive care unit utilization in the last 2 months of life in a subgroup of GBM patients assisted by the home care program were significantly lower than the control group who did not receive home assistance (16.7 vs. 38%, respectively; *p* < 0.001) (30). The authors, however, failed to report the impact of palliative home care program on QoL, quality of dying or caregiver burden.

#### Prevalence and Preference for Timing and Content of EOL Discussions

End-of-life discussions were poorly used in the included studies. Two studies reported that approximately 12–40% of pmBT patients did not have EOL discussions involving decisions regarding treatment preferences, health care proxy (HCP), palliative care consultation, hospice discussion, and resuscitation wishes (18, 25).

Gofton et al. retrospectively evaluated timing and content of EOL discussions in pmBT patients who were admitted to hospital within 6 months of death (25). The authors found that of 43 deceased pmBT patients, potential admission to a hospice was discussed in 38 patients (88%), a HCP was appointed in 33 patients (77%) and 28 patients (65%) had a do-not-resuscitate (DNR) order. Hospice discussions were initiated at a median of 39 days before death, and DNR orders were filled in at a median of 41 days before death. There was a wide degree of variation in the timing of EOL discussions such as hospice planning (1–140 days) prior to death and resuscitation wishes (1–140 days) (25). Overall, discussions about treatment restrictions in pmBT patients were often initiated relatively close to death (25). Common EOL treatment decisions also concern hydration (87%), comfort care (82%), steroid interruption (45%) tube feeding (13%), palliative sedation (13%), and ADs (6%) (30).

#### AD Completion Rates and Nomination of Health Care Proxy

Rates of AD completion varied widely between countries. In a study examining EOL care in HGG patients conducted in three European countries, the authors reported significant contrast in written AD completion, including 46% in Dutch, 37% in British, and only 6% in Austrian patients (*p* < 0.001) (28). Overall, the findings from the included studies in this review showed that around 0–76% of pmBT patients had an AD completed (3, 5, 17, 18, 23, 26, 28, 30, 31, 34). The nomination of health care proxies ranged from 8 to 77% among the studies (3, 22, 26).

#### Use of Palliative Care and Hospice, and Place of Death

The use of palliative care in pmBT is widely discussed in the included studies (3, 21, 24, 25, 27, 30, 31, 33). Collins et al. examined the clinical presentation and patterns of care for short-term pmBT survivors (21). The authors found that 22% of short-term survivors (those who died during initial admission) did not have contact with palliative care. Of the 23% (*n* = 264) of patients who survived the diagnosis admission but died within 120 days later, only 12% had a palliative care consultation for appropriate planning and support during their diagnosis admission. Surprisingly, the majority (38%) died in a palliative care bed/inpatient hospice, 32% died at home, and 30% in an acute hospital (21). It was suggested that timely referral to palliative care at the initial point of discharge may have facilitated death at home or in a more appropriate and cost-efficient hospital setting. This study was of retrospective design and did not evaluate other factors predicting shorter survival rates. In another study, Heese et al. retrospectively assessed the palliative care involvement in EOL support of pmBT patients and found that only 21.3% of patients received care from physicians who specialized in palliative medicine in their final 4 weeks prior to death (27).

A study conducted in Australia found that pmBT patients who received any palliative care in the last 120 days before death were more likely to die out of hospital (25). Pace et al. evaluating a pilot program of comprehensive palliative neurological home care for BT patients (30), showed a subgroup of GBM patients (*n* = 425) who were assisted by the palliative home care program had significantly lower hospital readmission rates, ICU utilization in the last 2 months of life compared to the control group who did not receive home assistance (16.7% vs. 38% respectively; *p* < 0.001) (30).

Similarly, there was variability in the reported POD among different countries. Study findings suggested that the reported rate of dying at home were 21–82% in USA (22, 25), 26–32% in Australia (21, 33), 40–66% in The Netherlands (5, 18, 28, 29, 31, 32), 53–65% in Italy (3, 30, 31), 16–33% in UK (6, 24), and approximately 23–48% in Germany (26, 27). Overall, approximately 8–33% of pmBT patients were reported dying in inpatient hospice (5, 21, 27–31, 33) or with affiliated hospice support (22, 25).

#### Patient/Surrogate Confidence and Congruence in EOL Decision-Making

Patient confidence in ACP decision-making was shown to be associated with various factors. El-Jawahri et al. in a RCT showed that patient confidence in EOL decision-making and ACP increased when a video support decision-making tool was used (17). The mean uncertainty score regarding decision-making (range, 3–15; higher score indicating less uncertainty) was significantly higher in the intervention group (with additional video support) compared to controls with verbal support only (13.7 vs. 11.5; *p* < 0.002) (17), with video arm participants significantly preferring comfort care (*p* < 0.0001). Approximately 82.6% in the video intervention arm in this study reported being very comfortable watching the video (17).

Sizoo et al. retrospectively assessed the patients’ relatives concerning EOL phase of pmBT patients involving health-related QOL, decision-making, place and quality of EOL care and dying with dignity (32). The authors found that higher satisfaction rates with the physician, fewer transitions between health-care settings in the EOL phase, ability to communicate EOL decisions, and more frequently dying at preferred POD were associated with a dignified death (32). This study, however, had various methodological flaws including retrospective design, proxy ratings with recall bias, and small sample size.

Various studies used congruence with patient wishes as an outcome measure. The findings suggested that completion of an AD is not often sufficient to ensure congruence with patients’ wishes for EOL care. In a study examining EOL care in pmBT patients in three European countries, physicians made decisions that were not in accordance with patients’ earlier expressed preferences, was reported in 10% of patients (28). The authors also found that the actual POD was in accordance with preferred POD only in 67% of Dutch, 56% of the Austrian and 39% of the British patients (*p* = 0.014), likely reflecting health care system differences toward EOL care (28).

Another retrospective survey of physicians in the Netherlands examining the EOL preferences of patients and congruence with physician decisions showed that in 40% of pmBT patients, physicians were unaware of patients’ EOL preferences, even though several had an AD according to their relatives (18). The authors found that these preferences were related to life-prolonging treatment preferences, admission to hospital, palliative sedation or euthanasia. The authors also indicated that there was incongruence between physician and patients’ EOL wishes with decisions made against patient’s wishes in a small proportion of patients (18).

#### Patient–Clinician Communication and Interaction Quality, Patient and Family Well-Being, QoL, and Satisfaction

Four studies found a significant beneficial effect of ACP on patient-clinician communication and interaction quality (28, 30–32). Sizoo et al. assessed the proportion of pmBT patients dying with dignity as perceived by their relatives (32). Factors most predictive of a dignified death included higher satisfaction rates with the physician, the ability to communicate EOL decisions and the absence of transitions between healthcare settings (32). These findings were consistent with another cross-national study comparing EOL care in pmBT patients in three European countries (28). Good quality of care (QOC) during the last 3 months prior to deaths was shown to be associated with effective treatment of physical symptoms and satisfaction with information provided (28). A home neuro-oncological palliative care program also had a positive impact on perception of QOC through communication and psychological support offering ACP and EOL care discussions perceived by caregivers (30, 31).

The review identified a limited number of studies examining the outcomes of ACP on patient or family well-being, QoL, and satisfaction. Caregiver QOL was generally rated low in the context of pmBT patients’ illness trajectory and this did not differ between caregivers of patients, who died at home (40%) or in hospital (46%) (5). One study examining the EOL phase of pmBT patients found that with those patients who died with dignity, EOL decisions were more often explicitly discussed and relatives were more satisfied with physician(s) providing EOL care (32). The authors report that pmBT patients who died most often with dignity were those who died at home (83%), compared to those who died in other health care settings including hospice (71%), hospital (63%), and nursing home (50%) (32). This study had some limitations due to retrospective design, recall bias based on relatives’ information and small sample size.

#### Hospital Utilization and Mortality Rates

Hospital utilization is frequently measured as an economic marker of health care costs. Hospital utilization in pmBT patients, in terms of POD, reported among the studies varied between different countries and ranged from 7 to 46% (5, 6, 18, 21, 26–33).

Two studies assessed hospital utilization as an outcome measure (21, 30). Collins et al. examined the patterns of care for shorter term survivors of pmBT patients in Australia (21). The authors divided these patients into Group 1 (19%, *n* = 218) (died during diagnosis admission), Group 2 (23%, *n* = 264) (survived diagnosis admission but died within 120 days from diagnosis) and Group 3 (58%, *n* = 678) (died thereafter) (21). As expected, Group 1 who had higher severity of illness burden and older patients tend to have higher hospital utilization. Group 1 comprised a greater proportion of patients aged over 75 years (*p* = 0.015), more patients with multifocal tumor (*p* = 0.001), and with more comorbidities (*p* = 0.001). These patients (Group 1) have higher total bed days per patient (*p* < 0.001), greater emergency department use (*p* = 0.001), greater ICU use (*p* = 0.001) and lower rates of resection (*p* < 0.001) (21). Another study demonstrated that in a subgroup of GBM patients, a home care neuro-oncological program, which included palliative ACP, significantly reduced hospital readmissions rates and ICU utilization in last 2 months of life compared to their counterparts without this service (16.7 vs. 38%, respectively; *p* < 0.001) (30). Furthermore, there was a significant difference in cost for the care delivered during hospital stays ($9990 vs. $76,000; *p* < 0.001) (30).

None of the studies assessed mortality outcomes associated with ACP, for example, whether or not ACP was associated with a difference in mortality rates in pmBT patients.

## Discussion

This systematic review provides an evidence-based overview of ACP in pmBT patients, assimilating published literature for currently available evidence by including both quantitative and qualitative studies. The review highlights the lack of robust, methodologically strong studies evaluating ACP in this population, with very few ACP interventions trialed to date. Due to the heterogeneity of the studies, best evidence synthesis was performed using a narrative approach. Only one RCT was found, which showed a positive effect in enhancing ACP information delivery to pmBT patients (17). The majority of the included studies in this review were retrospective in design, making it difficult to assess cause and effect of ACP interventions, and how to effectively conduct ACP in this population. These studies focused on EOL issues in pmBT patients, patterns of care, decision-making and palliative care support. The use of ACP also varied amongst countries with the lack of consistency of use and not being constant across the various contexts of care. Despite these limitations, the findings suggest some beneficial effects of ACP in pmBT, which include dignified death, lower hospital utilization and higher carer satisfaction.

This review highlights various issues in this area. First, there were limited number of studies examining ACP discussions in pmBT patients (18, 25) and, hence, it is difficult to draw conclusions on the existing rates of EOL discussions involving various aspects of ACP in pmBT patients. The findings also suggest that pmBT patients’ awareness of their prognosis varies considerably, with 40% being unaware of it (3, 35). The rates of AD completion ranged largely from none to 76% of patients, varying between different countries (3–5, 18). Although the presence of an AD is aimed at improving EOL care by involving an understanding between physicians and family members with regards to patients’ wishes and patients dying more often at preferred POD with less caregiver burden (10), AD completion was not always found effective. Congruence with patient wishes and EOL discussions was an interesting outcome measure noted in this review. In Europe, 10% of patients earlier expressed preferences were not in accordance with physicians’ decisions (28). Another study also reported that in 40% of patients, physicians were unaware of patients’ AD (18). There are many factors that impact on the inefficiency of these ACP discussions, and processes are often ineffective due to poor patient–physician communication and patients’ lack of sufficient medical knowledge to engage in these discussions. Furthermore, health professionals may differ in the interpretation of the patient’s current preference and may not always be reflected in their previous AD. Regular evaluation of documented AD is, therefore, advisable (36), along with clear communication with patients and surrogate decision-makers during ACP discussions regarding future wishes and treatment preferences. These goals should be reviewed over time as they may change depending on illness trajectory (18).

Second, there is limited data to demonstrate the appropriate timing of EOL discussions in patients with pmBT. The findings suggest a wide degree of variation in the timing of EOL discussions in pmBT patients such as hospice planning prior to death and resuscitation wishes (25). One study stated that 52% of patients were incompetent to assess their own situation in the last weeks of life due to cognitive disturbances, aphasia and/or delirium (25), with another 33% of patients who had lost their competence during the last week of life due to reduced consciousness (25). With a high chance of progressive functional and cognitive decline in pmBT patients and given the importance placed on mental competence in determining their QoL (18), timely and early discussions regarding GOC and treatment in EOL phase including ACP are encouraged (9, 18, 25). This will allow increased patient autonomy and patients to have an active shared treatment decision-making process with their physicians and family, resulting in higher QoL, increased survival rates and less caregiver distress (10). Authors suggest that the most frequent EOL decisions within the last 4 weeks of life in pmBT patients tend to focus upon hydration, nutrition, steroid interruption, AD, and palliative sedation (3).

Third, the effect of ACP in pmBT patients and their caregivers on confidence, well-being, QoL, and satisfaction were surprisingly not avidly evaluated. On study reported that an additional intervention including an ACP video had a positive impact on patients in preferring comfort care and avoiding CPR, with improved EOL decision-making (17). Studies evaluating caregivers and family perspectives found mixed results. One study found that although patient and caregivers had expressed their preferences for POD, the majority (68%) were not able to fulfill their preferences (5). For patients who died a dignified death, EOL decisions were more often explicitly discussed. Furthermore, caregivers were more satisfied with physician(s) providing EOL care (32), as they perceived that their loved one died a dignified death.

Place of death has also been commonly evaluated. Consistent with other cancer populations, most terminally ill pmBT patients prefer to be at home during the EOL phase (37, 38). However, the preferred POD may change over time and the actual POD may not be in line with patient’s preferences. There is a wide variability in POD between different countries, likely reflecting differences in feasibility of home care, use of hospices, different health models of care, and access to healthcare services, such as palliative care support.

Primary malignant brain tumor patients have diverse and fluctuating symptoms, and their needs differ from other palliative patients. Management of these complex patients should ideally performed by practitioners with expertise in supportive care of terminal neurological conditions. A neuropalliative model is an important example of a comprehensive approach to ACP delivery, seeking to improve QoL of patients and their families, as well as to address physical, psychosocial and spiritual needs (39, 40). The ultimate aim is to provide better care to those who need it most at the appropriate time, and to facilitate ACP discussions. The timeliness of referral to palliative care continues to be of great interest to healthcare professionals involved in the care of pmBT patients, however, the service remains under-utilized or continues to be initiated sporadically (21, 24, 25), and often used late in the cancer trajectory. To date, only a limited number of studies have evaluated early palliative care in conjunction with standard therapies for pmBT patients (41). This systematic review found only one moderate quality study that adopted a comprehensive approach to ACP delivery, including a pilot program of comprehensive palliative home care for pmBT patients (30). Consistent with the neuropalliative care framework, this model focused on patient’s needs for care in all stages of disease, aiming to support the families, deliver ACP information, and was shown to reduce economic costs for care delivered, and rehospitalization rates (30).There were no other studies which directly examined the cost-effectiveness for treatments involving ACP and mortality outcomes in this population.

There are several limitations in methodology and completeness of the retrieved literature. Despite the comprehensive search using extended range of terms to capture the relevant literature, the search strategy principally encompassed only cited literature. Furthermore, the only reference lists within the relevant articles was searched for other possible articles missed in electronic searches, this may have introduced a reference bias and may have missed some relevant articles, including unpublished trials. Finally, though the CASP approach used to appraise studies is a robust system for evaluating various trial-based evidence, its sensitivity is still debatable (42). As studies identified in this review were of mixed methods, CASP was appropriate tool. As aforementioned, pooling data for meta-analyses to make meaningful statements was not possible. The generalizability of the results is limited, due to heterogeneity in study designs, study settings, interventions used, participant recruitment processes, and outcomes measures used. Furthermore, generalizability of results to different countries and healthcare systems is limited as studies were conducted predominantly in USA and Europe, with varied healthcare systems.

In conclusion, this systematic review highlights sparse literature and lack of high-quality studies examining ACP in pmBT patients. Assimilation of data from existing studies was difficult due to heterogeneity amongst studies and their findings. The gap in current research should not be interpreted as ineffectiveness of ACP in this population, as ACP is important for pmBT patients where the risk of cognitive impairment and losing decision-making capacity are high. Overall, this review highlights the increasing awareness of ACP in pmBT patients for effective management in illness trajectory, particularly in terminal phase. ACP is an important element in improving EOL care of pmBT patients and needs to be considered for improved and/or shared decision-making by patients and family, increased patient and family satisfaction, improved physician and patient relationship, and enhanced QOL of the patient. Further high quality studies, especially RCTs, are needed to ascertain most effective types of ACP interventions in HGG population, and outcome measures could also be potentially standardized after evaluation in consensus meetings to overcome heterogeneity of study outcome measures.

## Author Contributions

KS, FK, CV, and BA participated in discussions to conceive the study. KS and BA participated in the analysis of results. KS participated in writing the manuscript, and all authors read and approved the final manuscript.

## Conflict of Interest Statement

The authors declare that the research was conducted in the absence of any commercial or financial relationships that could be construed as a potential conflict of interest.

## Abbreviations

ACP, advance care planning; AD, advance directive; BT, brain tumors; CASP, Critical Appraisal Skills Program; EOL, end-of-life; GBM, glioblastoma multiforme; pmBT, primary malignant brain tumor; QOC, quality of care; QoL, quality of life; WHO, World Health Organization.
